# Potential risk groups and psychological, psychosocial, and health behavioral predictors of pharmacological neuroenhancement among university students in Germany

**DOI:** 10.1038/s41598-022-04891-y

**Published:** 2022-01-18

**Authors:** Sebastian Heller, Ana Nanette Tibubos, Thilo A. Hoff, Antonia M. Werner, Jennifer L. Reichel, Lina M. Mülder, Markus Schäfer, Daniel Pfirrmann, Birgit Stark, Thomas Rigotti, Perikles Simon, Manfred E. Beutel, Stephan Letzel, Pavel Dietz

**Affiliations:** 1 Institute of Occupational, Social and Environmental Medicine, University Medical Center of the University of Mainz, Mainz, Germany; 2 Diagnostics in Healthcare and E-Health, University of Trier, Trier, Germany; 3 Department of Psychosomatic Medicine and Psychotherapy, University Medical Center of the University of Mainz, Mainz, Germany; 4 Department of Work, Organizational, and Business Psychology, Institute for Psychology, Johannes Gutenberg University, Mainz, Germany; 5 Department of Communication, Johannes Gutenberg University, Mainz, Germany; 6 Department of Sports Medicine, Rehabilitation and Disease Prevention, Institute of Sport Science, Johannes Gutenberg University, Mainz, Germany

**Keywords:** Psychology, Risk factors

## Abstract

Aiming to develop and implement intervention strategies targeting pharmacological neuroenhancement (PN) among university students more specifically, we (1) assessed the prevalence of PN among German university students, (2) identified potential sociodemographic and study-related risk groups, and (3) investigated sociodemographic, psychological, study-related psychosocial, general psychosocial and health behavior related factors predicting the 12-month prevalence of PN. Therefore, a cross-sectional online survey was administered to students of the University of Mainz, Germany. A binary logistic regression with stepwise inclusion of the five variable groups was performed to predict PN. A total number of 4351 students out of 31,213 registered students (13.9%) participated in the survey, of which *N* = 3984 answered the question concerning PN. Of these, 10.4% had used one substance for PN at least once in the past 12 months. The regression analysis revealed 13 variables that were significantly related to the 12-month prevalence of PN. Specifically, the group of health behavior related variables showed the strongest relationship with PN. Therefore, an approach to the prevention of PN should be multifactorial so that it addresses social conditions, as well as education on substance use and healthy behaviors in terms of non-pharmacological strategies as alternatives of PN.

## Introduction

The term “pharmacological neuroenhancement” (PN), also called “pharmacological cognitive enhancement”, is generally defined as the use of illicit or prescription drugs by healthy individuals for cognitive-enhancing purposes^1–3^, such as enhancing alertness, attention, concentration, memory, and also mood^4,5^. According to this definition, the so called soft neuroenhancers (e.g. energy drinks, caffeine tablets, ginkgo biloba) are not included. There are many inconsistencies and differences in the definition^6,7^, but a full discussion of these would go beyond the scope of this research.

In the past decade, a considerable number of studies demonstrated that PN is not uncommon in western populations. For example, epidemiological studies from western Europe and the United States reported 12-month prevalences of PN in the general population between 2.1 and 6.6%^8–10^. Most often named reasons for PN (78.2%) were improving alertness, enhancing concentration, or help to study^8^. With regard to specific occupational settings in Germany, such as surgeons and economists, the lifetime prevalence of PN has been reported to range between 8.9 and 19%^4,5^.

A very well-examined group with an increased risk for PN are university students. For example, a large study, comparing the non-medical use of prescription stimulants between US college students and respondents of the same age not enrolled in college (*N* = 15,454), showed that college students used prescription stimulants more often (OR 1.28, 95% CI 1.05, 1.56) compared to non-students of the same age group^11^. Moreover, within a comprehensive review and meta-analysis, Benson et al.^12^ reported 12-month prevalences for the use of prescription stimulants between 5 and 35% among college students in the US, demonstrating large heterogeneity in the range of these prevalence rates. Studies among university students in Western Europe obtained results in a similar range. For example, lifetime prevalences for PN of 7.8% among Swiss (*N* = 6275)^13^, 3.2% among Norwegian (*N* = 9370)^14^, and 19.2% among British students (*N* = 506)^15^ were reported. The same tendencies appear among German university students, as 12-month prevalence estimates between 11.9^16^ and 20%^17^, assessed by indirect survey techniques, were reported.

From a public health point of view, the above-mentioned figures for the use of PN, especially in university students, are alarming because PN appears to be associated with physiological and psychological side effects, may increase mortality, and can lead to addiction^18–23^. Understanding the conditions and factors predicting PN, especially among the severely affected collective of university students, contributes to evidence-based planning of PN-prevention strategies because effective programs have to target factors related to PN. Therefore, potential correlates (factors that are associated) or determinants (factors with a causal relationship) of PN need to be investigated^24^. In this context, some research has already investigated potential variables being related to PN, such as sociodemographic aspects^16,17^, psychological factors, such as stress^25–28^ or specific demands and resources^29–32^, psychosocial factors, as well as health behavior related factors, such as health-related risk attitude^16^ or eating behavior^33^. Concerning psychosocial factors, it can be differentiated between ‘study-related psychosocial factors’ (factors that are only relevant for the specific collective of university students), such as perceived academic benefits^34–38^ and more ‘general psychosocial factors’ (factors that are not only relevant for the specific collective of university students), such as impulsiveness^39^.

Among this body of research, studies in university students examining the role of explanatory variables with an adequate sample size are rare. Furthermore, to the best of our knowledge, we are not aware of any study investigating the relation between PN and sociodemographic factors, psychological factors, study-related and general psychosocial factors, as well as health behavior related factors in one model. In addition, Faraone et al.^7^ made a strong claim that, because of limited data availability, and variations in describing the use of PN, more research is needed to identify potential risk groups of PN in order to develop effective prevention and treatment interventions.

To conclude, empirical studies addressing PN among university students are heterogeneous regarding their methodology and results^13–17,40,41^. Moreover, there is a considerable lack of knowledge regarding potential factors that might predict PN and regarding the identification of potential study-related risk groups. Therefore, within the present study, we addressed these issues, and (1) assessed the prevalence of PN among German university students aiming to (2) identify potential sociodemographic and study-related risk groups, especially with regard to age, gender, field of study, semester, aspired degree, and (3) investigate factors related to PN by including sociodemographic factors, psychological factors, study-related and general psychosocial factors, as well as health behavior related factors in one stepwise regression model. This enables us to identify more general factors and specific variables that might be more or less strongly related to PN. These results are valuable to (4) develop and implement intervention strategies targeting PN among university students more specifically.

## Methods

### Study design and survey procedure

All students of the University of Mainz (31,213) were invited (using the university’s central mailing list) to a cross-sectional online health survey in summer term (June and July) 2019 as part of an ongoing project on health promotion among students (“Healthy Campus Mainz”). Reminder emails were sent four times. In an introduction at the beginning of the online questionnaire, the background and purpose of the study were shortly explained, followed by a statement that participation would be anonymous and voluntary. Informed consent was obtained at the beginning of the survey. A total number of 4351 students participated in the survey, demonstrating a response rate of 13.9% of the university’s total student population at that time. The survey was designed using the software Unipark. A mixed incentive strategy was chosen to reach a wide range of different people among the student population^42^. The main incentive was the following: “*If 5000 people complete the questionnaire, 1000€ will be donated to the child cancer aid of Mainz.*” This charity organization was chosen since it is directly linked to the topic of health. Throughout the whole survey implementation, the students were informed via reminder emails and social media about the current number of completed surveys to further promote participation. Besides this, a lottery of gift cards for local gastronomy providers and an online store functioned as monetary incentives. We included 13 gift cards for local gastronomy providers (7 × 24€ and 6 × 40€). In addition, we offered 15 gift cards for an online store (5 × 100€, 5 × 50€, and 5 × 20€). Approval to perform the study was given by the ethical committee of the Medical Association of Rhineland-Palatinate (application-number: 2019-14336). The study was performed in accordance with the Code of Ethics of the World Medical Association (Declaration of Helsinki) for experiments involving humans and the Ethical Principles and Guidelines for the Protection of Human Subjects of Research by the American Psychological Association (APA). Further information on the study design and the survey is provided by Reichel et al.^42^.

### Measures

The online survey covered a wide range of health-related topics containing approximately 270 items. Established and validated instruments were used whenever feasible and self-developed scales were used as little as possible. A list of all surveyed topics and items is given by Reichel et al.^42^. To predict PN for the present paper, 55 independent variables with regard to the research questions were selected. A list of the specific variables, scales, and items used for the present analyses, as well as the respective references and specific questions with answering options (for self-constructed items), is given in Supplementary Table 1. These 55 variables were classified into 5 different groups (according to the factor groups of current research, as described in the introduction): sociodemographic variables (14 variables, e.g. gender, age, semester, field of study), psychological variables (6 variables, e.g. depressive symptoms, emotional exhaustion), study-related psychosocial variables (17 variables, e.g. social support by fellow students, self-efficacy), general psychosocial variables (5 variables e.g. self-criticism, impulsiveness), and health behavior related variables (13 variables e.g. alcohol use, healthy diet, physical activity).

The prevalence of PN was assessed according to Dietz et al.^4^. The translated question regarding PN was: “Have you ever used the following substance/-s without medical necessity, for the purpose of enhancing your cognitive performance or to better handle your studies (not for reasons of enjoyment)?”. The following illicit or prescription drugs could be selected via multiple-choice, and for each drug via single-choice on a scale consisting of ‘never’, ‘within the last 30 days’, ‘within the last 12 months’, or ‘more than 12 months ago’: methylphenidate (e.g. Ritalin^®^), amphetamine preparation (e.g. Adderall^®^), atomoxetine (e.g. Strattera^®^), modafinil (e.g. Provigil^®^), ecstasy, ephedrine, cocaine, illicit amphetamines (e.g. Speed), crystal meth, cannabis, and ‘other substances’.

### Data analysis

Descriptive statistics are presented as means with standard deviations (SD) for continuous scaled variables and as numbers and percentages for non-continuous scaled variables. To analyze prevalence differences between sociodemographic and study-related groups, contingence-analyses of categorial variables were performed by means of Pearson chi-square (χ^2^) tests. Therefore, the continuous variable age was dichotomized at the median. Results on the prevalence of PN are given as percentages, numbers, and p values.

In order to enable the investigation of potential changes in the prevalence of PN in future studies, the 12-month prevalence instead of the lifetime prevalence was used for all further analysis. The 12-month prevalence of PN summarizes the number of participants who stated to have used at least one of the surveyed substances ‘within the last 12 months’ or ‘within the last 30 days’, while the lifetime prevalence reflects the number of participants who stated to have used at least one of the surveyed substances once in their life, summarizing the categories ‘more than 12 months ago’, ‘within the last 12 months’ or ‘within the last 30 days’.

Pretests, using Spearman correlation for continuous (Supplementary Table 2) and Pearson’s chi-square test for categorial variables (Supplementary Table 3), were performed for each of the 55 independent variables. Only variables that showed a significant association with PN in the pretest (*p* ≤ 0.001) were included into the regression analysis (see Supplementary Tables 2 and 3). A binary logistic regression with stepwise inclusion of the 5 variable groups was performed to predict PN. Therefore, the dependent variable PN was dichotomized (PN use within the last 12-months/never used PN). Here, the participants who answered ‘more than 12 months ago’ were excluded. Multicollinearity was checked with the help of a collinearity matrix and the variance inflation factor (VIF). Furthermore, an appropriate sample size of the regression model was determined by the criterion of 50 events per variable + 100. Using simulations, Bujang et al.^43^ revealed that this formula is valid to determine sample size of observational studies, independently of an observed effect size. Accordingly, for 55 variables, a minimum sample size of *n* = 2850 would be needed. In order to check for the robustness of results, the binary logistic regression model was cross-validated using an 80% random sample. Statistical analysis was performed using IBM SPSS Version 23 V5.

## Results

Of the total number of 4351 students who participated in the health survey, *N* = 3984 participants answered the question with regard to PN and were included in the analyses. Mean age of the sample was 23.8 years (SD = 4.3 years), and 71.3% (*n* = 2842) of the participants were female. Compared to the distribution of age and gender at the University of Mainz as a whole, the mean age was approximately representative (24.7 years was the mean age of the University’s whole student body at that time) and women were overrepresented by 12.3 percentage points. With regard to study-related characteristics, 16.3% (*n* = 650) of the participants were first-year students, 52.4% (*n* = 2086) were pursuing a bachelor’s degree, 21.2% (*n* = 844) for a master’s degree, 22.0% (*n* = 876) were aiming for a German state examination (e.g., law and medical students and students of teaching professions), and 3.5% (*n* = 139) were PhD students. All sociodemographic and study-related characteristics of the participants are presented in Table 1.

**Table 1:** Characteristics of the study sample and values for the 12-month prevalence of pharmacological neuroenhancement distributed for sociodemographic and study-related characteristics.

Variable	Value			*χ*^*2*^, *p,* V; categories with statistically significant differences between prevalence of ‘12-month PN users’
	Total sample	12-month PN users	PN non-users	
All, *n* (%)	3984 (100.0)	416 (10.4)	3384 (84.5)	
**Gender, *n* (%)**				
(a) Female	2842 (71.3)	263 (9.3)	2458 (86.5)	*χ*^*2*^ = 21.6, *p* < 0.001, V = 0.052; a-b
(b) Male	1110 (27.9)	146 (13.2)	903 (81.4)	
(c) Diverse	32 (0.8)	7 (21.9)	23 (71.9)	
**Age, range (mean ± SD)**	16–73 (23.8 ± 4.3)	18–52 (23.6 ± 3.9)	16–73 (23.7 ± 4.3)	
**Semester, range (mean ± SD)**	1–45 (7.2 ± 4.8)	1–24 (6.6 ± 4.5)	1–45 (7.2 ± 4.8)	
**Age**^**#**^				
(a) Younger or equal 23 years	2243 (56.4)	233 (10.4)	1929 (86.0)	*χ*^*2*^ = 12.1, *p* = 0.002, V = 0.055
(b) Older than 23 years	1737 (43.6)	182 (10.5)	1452 (83,6)	
**First year, n (%)**				
(a) Yes	650 (16.3)	79 (12.2)	554 (85.2)	*χ*^*2*^ = 9.1, *p* = 0.010, V = 0.049
(b) No	3228 (81.0)	320 (9.9)	2747 (85.1)	
**Field of study, *n* (%)**				
(a) STEM	720 (18.1)	65 (9.0)	628 (87.2)	*χ*^*2*^ = 22.6, *p* = 0.031, V = 0.053; a-b; b-f; e–f
(b) Social sciences, media or sport	717 (18.0)	89 (12.4)	598 (83.4)	
(c) Philosophy, humanities or cultural sciences	803 (20.2)	89 (11.1)	674 (83.9)	
(d) Medicine	528 (13.3)	51 (9.7)	439 (83.1)	
(e) Law or economics	512 (12.9)	63 (12.3)	432 (84.4)	
(f) Aspiring teachers	614 (15.4)	50 (8.1)	537 (87.5)	
**Degree, *n* (%)**				
(a) Bachelor	2086 (52.4)	254 (12.2)	1734 (83.1)	*χ*^*2*^ = 39.3, *p* = 0.025, V = 0.061; a-b; a-d; b-c; c-d
(b) Master	844 (21.2)	63 (7.5)	751 (89.0)	
(c) State examination	876 (22.0)	91 (10.4)	738 (84.2)	
(d) Doctorate	139 (3.5)	5 (3.6)	126 (90.6)	

‘12-month PN users’: respondents who used pharmacological neuroenhancement within the last 12 months; ‘PN non-users’: respondents who did not use pharmacological neuroenhancement within the last 12 months; alphabetic characters ‘a-b’; ‘a-d’; ‘b-c’; ‘c-d’ represent significant differences (*p* < 0.05) in the prevalence of ‘12-month PN users’ between respective categories of that variable. Note that the category ‘more than 12 month ago’ for the prevalence of pharmacological neuroenhancement (n = 184) is not presented in this table; SD: standard deviation; #: age dichotomized at the median; STEM: science, technology, engineering, and mathematics.

### Prevalence of PN and identification of potential risk groups

Among all participants, 15.1% (*n* = 600) had used one of the listed substances for PN at least once in their life (lifetime prevalence), and 10.4% (*n* = 416) at least once in the past 12 months (12-month prevalence). The most commonly used substance for PN was cannabis (7.2% 12-month prevalence; Table 2).

**Table 2:** Prevalences for the use of illicit or prescription drugs for pharmacological neuroenhancement among students at the University of Mainz (*n* = 3984).

Use of any surveyed substance	Never used	Used			
		Total responses	Within the last month	Within the last 12 months	More than 12 months ago
**Prescription and illicit drugs**					
Methylphenidate	97.1% (*n* = 3868)	2.9% (*n* = 115)	0.7% (*n* = 26)	0.7% (*n* = 28)	1.6% (*n* = 61)
Amphetamine preparation	99.6% (*n* = 3969)	0.4% (*n* = 14)	0.1% (*n* = 4)	0.1% (*n* = 3)	0.8% (*n* = 7)
Atomoxetine	99.8% (*n* = 3976)	0.2% (*n* = 7)	0.1% (*n* = 5)	< 0.1% (*n* = 1)	< 0.1% (*n* = 1)
Modafinil	99.5% (*n* = 3965)	0.5% (*n* = 18)	0.2% (*n* = 9)	0.1% (*n* = 4)	0.1% (*n* = 5)
Ecstasy (MDMA)	98.3% (*n* = 3917)	1.7% (*n* = 67)	0.3% (*n* = 13)	0.6% (*n* = 25)	0.7% (*n* = 29)
Ephedrine	99.6% (*n* = 3967)	0.4% (*n* = 15)	0.1% (*n* = 2)	0.2% (*n* = 6)	0.2% (*n* = 7)
Cocaine	98.7% (*n* = 3931)	1.3% (*n* = 53)	0.2% (*n* = 8)	0.4% (*n* = 17)	0.7% (*n* = 28)
Amphetamine	98.2% (*n* = 3914)	1.8% (*n* = 70)	0.4% (*n* = 17)	0.5% (*n* = 19)	0.9% (*n* = 34)
Crystal Meth	99.9% (*n* = 3980)	0.1% (*n* = 4)	< 0.1% (*n* = 1)	0.1% (*n* = 2)	< 0.1% (*n* = 1)
Cannabis	89.3% (*n* = 3557)	10.7% (*n* = 426)	3.6% (*n* = 142)	3.6% (*n* = 142)	3.6% (*n* = 142)
Other substances	96.9% (*n* = 3860)	3.1% (*n* = 124)	1.4% (*n* = 55)	0.9% (*n* = 36)	0.8% (*n* = 33)

Range of missing cases among drugs for pharmacological neuroenhancement = 1–2.

The 12-month prevalence of PN varied across sociodemographic and study-related groups. In male students, the prevalence was significantly higher (13.2%) compared to female students (9.3%, *p* < 0.001). First-year students showed a slightly higher 12-month prevalence (12.2%) compared to higher semester students (9.9%), and a significant difference was identified between bachelor (12.8%), and master (7.7%) students (*p* = 0.010 and *p* = 0.025, respectively). The 12-month prevalences for all sociodemographic and study-related variables are given in Table 1.

The 12-month prevalence of PN distributed for the different fields of study indicates that ‘aspiring teachers’ had a significantly (*p* = 0.031) lower prevalence of PN (8.1%) compared to students of ‘social sciences, media and sport’ (12.4%) and ‘law and economics’ (12.3%). Students of ‘STEM’ (science, technology, engineering, and mathematics) also had a significantly lower prevalence (9.0%) compared to ‘social sciences, media and sport’ (12.4%, Table 1).

### The relation between the 12-month prevalence of PN and sociodemographic, psychological, study-related psychosocial, general psychosocial, and health-behavioral factors

The sample size for pretests and binary logistic regression after the above-described protocol of dichotomization was *n* = 3800. Pretesting (Supplementary Tables 2 and 3) revealed 29 of 55 variables that were significantly associated with the 12-month prevalence of PN. Due to the number of variables, case processing of the logistic regression included *n* = 3608 cases into the analysis, demonstrating an appropriate size respectively events per variable according to Bujang et al.^43^ (as mentioned in the methods section). However, the first run of the regression analysis demonstrated that the variable “degree” had to be removed from the model, because it showed *p* values between 0.999 and 1 in all categories.

Subsequently, the overall model of the stepwise binary logistic regression was statistically significant, χ^2^ (40) = 490.590, *p* ≤ 0.001. Testing for multicollinearity revealed no collinearity of the chosen variables at all (with an average VIF of 1.6, the highest was 3.5, and the lowest 1.0). The stepwise regression showed changes in the explained variance of the model by stepwise inclusion of each variable group. The explained variance (Nagelkerke *R*^2^) in the last step of the 5-step-model was 25.7%, and it correctly classified 89.5% of cases. In the previous steps, the explained variance of the model increased from 1.0% in the 1st to 10.0% in the 4th step (see Supplementary Table 4). Cross-validation with an 80% random sample showed robustness of the regression model (see Supplementary Table 5). The last (5th) step of the regression revealed 13 independent variables that were significantly (*p* ≤ 0.05) related to the 12-month prevalence of PN (Table 3): ‘gender—male’ (out of sociodemographic variables), ‘depressive symptoms’ (out of psychological variables), ‘absenteeism’, ‘social support by fellow students’ (out of study-related psychosocial variables), ‘self-criticism’ (out of general psychosocial variables), and ‘healthy diet’, ‘moderate-vigorous physical activity’, ‘alcohol use’, ‘smoking cigarettes’, ‘coffee’, ‘caffeine tablets’, ‘coke’, and ‘ginkgo biloba’ (out of health-behavioral variables). For descriptive statistics of these significant predictors, please see Supplementary Table 6. The other 15 of the 28 independent variables that had been selected and showed a significant relation (*p* ≤ 0.001) to PN in pretests (as described earlier), were not significantly related to the 12-month prevalence of PN in the last step of the binary logistic regression model (see Supplementary Table 4).

**Table 3:** Significant predictors of the 12-month prevalence of pharmacological neuroenhancement in a binary logistic regression analysis with stepwise inclusion of the 5 independent variable groups: sociodemographic, psychological, study-related psychosocial, general psychosocial and health behavior related variables.

	OR (95% CI); ‘reference category’	*p*
**Sociodemographic variables**		
Gender—male	1.387 (1.052–1.827); ‘female’	0.02
**Psychological variables**		
Depressive symptoms	1.086 (1.044–1.129)	< 0.001
**Study-related psychosocial variables**		
Absenteeism	1.022 (1.005–1.040)	0.01
Social support (fellow students)	0.830 (0.721–0.955)	0.009
**General psychosocial variables**		
Self-criticism	0.955 (0.926–0.986)	0.004
**Health behavior variables**		
Healthy diet	1.264 (1.117–1.431)	< 0.001
Moderate-vigorous physical activity (min/day)	1.001 (1.000–1.002)	0.011
Alcohol use	1.612 (1.243– 2.092)	< 0.001
Smoking cigarettes	1.468 (1.349–1.597)	< 0.001
Coffee		
Within the last 30 days	1.531 (1.114–2.106); ‘never’	0.009
Caffeine tablets		
Within the last 30 days	4.235 (2.552–7.027); ‘never’	< 0.001
Within the last 12 months	1.763 (1.011–3.073); ‘never’	0.046
More than 12 months ago	2.117 (1.416–3.164); ‘never’	< 0.001
Coke		
Within the last 30 days	1.669 (1.255–2.221); ‘never’	< 0.001
Within the last 12 months	2.267 (1.587–3.239); ‘never’	< 0.001
Ginkgo biloba		
Within the last 30 days	2.587 (1.164–5.747); ‘never’	0.02

Observed cases:* n* = 3608; *R*^*2*^ = 0.257; *χ*^*2*^ (40) = 490.590; *p* < 0.001; please find results of the binary logistic regression model for all included (significant and non-significant) variables in Supplementary Table 4.

Male students had a higher likelihood of PN ‘within the last 12 months’ compared to female students. ‘Depressive symptoms’ was positively related to the 12-month prevalence of PN. Likewise, the more the respondents stayed away from their study events regularly (i.e. absenteeism) and the more excessive they rated their demands, the higher was the likelihood of PN within the last 12 months. Negatively related variables were ‘social support by fellow students’ and ‘self-criticism’: the greater their expression, the less likely was PN within the last 12 months. Among health behavior variables, sticking to a healthy diet, extent of moderate-vigorous physical activity, risky alcohol use, currently smoking cigarettes, consumption of coffee, caffeine tablets, coke, and ginkgo biloba were positively related to the 12-month prevalence of PN.

## Discussion

The present results demonstrate that the 12-month prevalence of PN among German university students differs in regard to sociodemographic and study-related groups, and that a model with groups of sociodemographic, psychological, psychosocial, and health behavior related variables is suitable to explain the 12-month prevalence of PN. In that model, specifically the group of health behavior related variables shows the strongest association with PN.

Referring to the first research question, namely to assess the prevalence of PN among university students, the overall 12-month prevalence for PN was 10.4%. This prevalence is approximately in the middle of the reported prevalences for university students from western European countries^13–17,40^. As stated above, these differences among reported prevalences may be caused by various methodological aspects (e.g. definition of PN, survey technique or period of reported prevalence).

With regard to the second research question, namely the identification of potential risk groups for PN in the collective of university students, male students showed a significantly higher risk for PN compared to female students. This finding is in line with previous studies^12,44,45^, showing that substance use appears to be more common in males than in females in different populations. With regard to study-related risk groups, first-year students and bachelor students were of increased risk for PN. This implies that PN is practiced early during studies, confirming the findings of Dietz et al.^17^. Furthermore, the prevalence of PN varied between different fields of studies: especially students of ‘social sciences, media and sport’ had a higher risk for PN compared to students from other fields of study. A possible explanation may be that the use of nutritional supplements (e.g., vitamins, minerals, herbals, caffeine, or creatine) is common in the field of sports and discussed to provide a gateway to the use of illicit drugs^46–49^.

In view of the third research question, namely the investigation of the explanatory role of sociodemographic, psychological, study-related and general psychosocial, and health-behavioral variables, gender was the only significant sociodemographic variable in the regression model. This is in accordance with the results of the contingency-analysis (second research question), that male students had a higher likelihood of PN within the last 12 months compared to female students. Among the group of psychological variables, ‘depressive symptoms’ showed a small positive association with PN. Surprisingly, other psychological variables such as ‘general anxiety’, ‘social anxiety’ or ‘loneliness’ were not significantly related to PN in our model, although previous studies reported associations^44,50–52^. In contrast to our study, these studies investigated the association of the different psychological variables and PN more isolated and not in a large model, as we did in the present study. Concerning the study-related and general psychosocial variables, a small protective effect of ‘social support by fellow students’ and ‘self-criticism’ for the use of PN was revealed. To the best of our knowledge, these variables were not investigated before when predicting PN. In this context, more ‘social support by fellow students’ also could be associated with more organized studying or less competition and therefore reduce stress and the subjective need of PN to increase academic performance^26^. The preventive effect of ‘self-criticism’ could be due to higher personal standards and perfectionism of more self-critical individuals^53^, and therefore, they might rather try to reach their academic performance goals by themselves and without external help through PN. Likewise, a potential explanation for ‘absenteeism’ predicting PN is that staying away from lectures increases the pressure to catch up on learning material and to successfully pass an exam so that PN may be used to cope with these demands. In the context of these results, it is also plausible that PN is a coping strategy in a vicious cycle of depression, missing in lectures, low social support, and maybe other forms of substance use or self-endangering behaviors. In general, self-endangering behaviors (e.g. presenteeism or prolonging working hours) represent maladaptive coping strategies and have previously been associated with higher quantitative demands and autonomy (in a u-shaped connection) among students^54^.

Interestingly, the group of health behavior related variables (healthy diet, extent of moderate-vigorous physical activity, alcohol use, smoking cigarettes, and using soft neuroenhancing substances) contributed the most to the explanation of PN in our model. Surprisingly, although ‘healthy diet’ appears as a health-protective factor for several issues^55^, the present results indicate that ‘healthy diet’ (for definition, see Supplementary Table 1) increases the likelihood of PN. A possible explanation could be that a healthy diet is also associated with cognitive benefits^56^, and consequently, it could be used as a co-strategy for neuroenhancement. However, sticking to a healthy diet might also be biased by subjective assessment of one’s own diet and therefore may be an indicator of restrictive diets or eating disorders that have been investigated to positively relate to PN^33,57^. The extent of moderate-vigorous physical activity showed a very small positive association with PN. This might be linked with the higher prevalence of PN in the cohort of students from the faculty of ‘social sciences, media and sport’. Furthermore, PN was strongly associated with other forms of drug involvement (alcohol use, smoking cigarettes). Whereas most of the final model’s odds ratios were small, for the consumption of soft neuroenhancers (coffee, caffeine tablets, coke, and ginkgo biloba), we obtained medium effects on the likelihood of PN within the past 12 months. While other forms of drug use, like drinking and smoking, may be a reason for decreased academic performance^58^ and reinforce the apparent necessity of PN, these soft neuroenhancers may provide a gateway to PN^1,49,59^. Especially for the use of caffeine tablets, relations to PN were already stated by other studies^16,60^. But besides these potential dangers of shifting from legal to illicit substance use, the consumption of high dosages of caffeine is also associated with adverse health-effects^61–64^. However, it should be noted that this block of health behavior related variables includes other forms of substance use, and since PN also represents a form of substance use, a greater explanation of variance by this step seems plausible.

With regard to research question four, in order to develop health promotion and prevention programs of high quality, such programs should be evidence-based. Since we revealed a higher prevalence of PN in first-year and bachelor students, prevention of PN should start early during studies or even at the end of school. Therefore, more research on the prevalence of PN among pupils, especially in graduation classes, would also be beneficial. Consequently, given that the present results show that PN is predicted particularly by the use of soft neuroenhancers, strategies tailored to educate on the use and effects of these substances may also help to prevent the more harmful use of drugs for the same purpose. Prevention strategies on general consumption of intoxicants, like drinking and smoking, may also decrease the risk of engaging in PN, since this study demonstrated the contributing effect of risky alcohol consumption, or smoking cigarettes. Moreover, since some individuals seem to use PN without critical reflection of potential consequences^38^, students should be educated about the limited efficacy of PN in healthy individuals^65,66^ and that PN is not associated with better marks or increased academic performance^67^. Additionally, more research should focus on the role of social support in the context of PN because cultivating and developing social support such as networks of communication and mutual obligation acts as a great resource^68–70^, not only with respect to the prevention of PN. Interventions that are targeted at the risk groups and use a multifactorial approach could lead to effective prevention of PN in future. Such a multifactorial prevention approach should therefore address social conditions, and educate on substance use as well as on healthy behaviors to increase cognitive performance—such as nutrition, physical activity, and mindfulness^56,71–73^—and how to adopt these behaviors as habits.

With regard to potential limitations of the present study, one could argue that whenever sensitive topics are studied, participants often react in a way that negatively affects the validity of study results (underreporting and non-responding) due to hesitating to provide compromising information about themselves^74,75^. Therefore, other studies used indirect survey techniques such as the randomized response technique (RRT) for the assessment of socially desirable questions such as PN^76,77^. Since the 12-month prevalence of PN of 10.4% in our survey is comparable to those of RRT-surveys^16^, it may imply that an online poll is subjectively perceived as anonymous and private and therefore provides comparable results to RRTs. Another potential limitation might be the relatively low response rate of 13.9%. However, it was slightly higher compared to university student health surveys of a similar extent that, for example, had response rates of 10%^78^ and 9%^79^. Possibly, the promotion of the survey via multiple channels and the differentiated incentive strategy could be an explanation for this. Compared to other health surveys at German universities^80,81^, the total sample size of our health survey is also quite large, despite the length of the survey. Besides, it has to be noted that some other studies do not report completion or response rates, which makes comparisons difficult.

In general, our online survey aimed to reach all students of Mainz University. Nevertheless, as participation was voluntary, we cannot exclude a certain selection bias in our sample. For example, health interested students and students of health-related disciplines might be more likely to participate in a health survey. Moreover, the survey was available only for German-speaking students. Because of this potential bias, the results regarding the prevalence of PN have to be interpreted with caution. Nevertheless, a strength lies in the robust associations on individual level. It also has to be noted that our study had a cross-sectional design, and therefore, no causality of the analyzed conditions can be confirmed.

## Conclusion

This study reveals that the 12-month prevalence of PN among German university students differs in regard to sociodemographic and study-related groups, with specific risk groups being males, first year students, and the study fields of ‘social sciences, media or sport’, and ‘law or economics’. Therefore, future studies should be performed with respect to the prevalence of PN in school graduation classes, and prevention of PN should start early during studies or even at the end of school. This study further reveals that a model with groups of sociodemographic, psychological, psychosocial and health behavior related variables is suitable to explain the 12-month prevalence of PN. In that model, specifically the group of health behavior variables has the strongest influence on the explained variance of PN. Therefore, an approach to the prevention of PN should be multifactorial so that it addresses social conditions, as well as education on substance use and healthy behaviors to increase cognitive performance and cope with stress. Students should be aware of and be able to habitually implement non-pharmacological coping-strategies^82^ that can help to increase cognitive performance and mood, such as physical activity^83^, nutrition, and relaxing or mindfulness techniques.

## Supplementary Information

Supplementary Information 1.

## Data Availability

The datasets generated and analyzed during the current study are stored on the server of the University Medical Center of the University of Mainz (European server) and are available from the corresponding author on reasonable request.
