# Supplementary material for: Potential risk groups and psychological, psychosocial, and health behavioral predictors of pharmacological neuroenhancement among university students in Germany
Source: Sci Rep. 2022 Jan 18;12:937. doi: 10.1038/s41598-022-04891-y (PMC8766436; doi:10.1038/s41598-022-04891-y)
Supplement: Supplementary file 1 — Supplementary Information 1. [file 41598_2022_4891_MOESM1_ESM.pdf]

Potential risk groups and psychological, psychosocial, and health behavioral predictors of pharmacological neuroenhancement among university students in Germany

Sebastian Heller[1], Ana Nanette Tibubos[2], Thilo A. Hoff[1], Antonia M. Werner[3], Jennifer L. Reichel[1], Lina M. Mülder[4], Markus Schäfer[5], Daniel Pfirrmann[6], Birgit Stark[5], Thomas Rigotti[4], Perikles Simon[6], Manfred E. Beutel[3], Stephan Letzel[1], Pavel Dietz[1*]

[1]Institute of Occupational, Social and Environmental Medicine, University Medical Center of the University of Mainz, Germany

[2]Diagnostics in Healthcare and E-Health, University of Trier, Germany

[3]Department of Psychosomatic Medicine and Psychotherapy, University Medical Center of the University of Mainz, Germany

[4]Department of Work, Organizational, and Business Psychology, Institute for Psychology, Johannes Gutenberg University Mainz, Germany

[5]Department of Communication, Johannes Gutenberg University Mainz, Germany

[6]Department of Sports Medicine, Rehabilitation and Disease Prevention, Institute of Sport Science, Johannes Gutenberg University Mainz, Germany

[*]corresponding author

Mail: pdietz@uni-mainz.de

Phone: +49 (0)6131 17 – 9339

Dimension	Variable (count)	Scale and/or Reference	Items	'Questions of self-constructed items', *answering options*
**Sociodemographic variables**	Gender (1)	Self-constructed items	1	'Your gender:' *male (1) / female (2) / diverse (3)*
	Degree (1)		1	'Which degree do you pursue with your current study?' *Drop-down list of all degrees at the university of Mainz*
	Age (1)		1	'Your age in years:'
	Relationship status (1)		1	'What is your relationship status?' *Single (1) / Partnership in one household (2) / Partnership with separate living conditions (3)*
	Financial status (1)		1	'What is the amount of money you monthly have at your disposal in average?'
	Employment (1)		1	'Are you currently employed?' *No (1) / Yes, full-time (2) / Yes, regularly part-time (3) / Yes, marginally or irregularly employed (4)*
	Support by parents/relatives (1)		1	'How do you finance your study?' *Support by parents or relatives: Yes / No*
	Parents' educational background (1)		1	'Please select the highest educational attainment of your mother or your father (the highest of both):' *List of attainments, also including ,'other attainment'*
	Migration background (1)		1	'Are both of your parents born in the today's area of Germany?' *Yes (1) / No (2) / Don't know (3) / No information (4)*
	Personal migration experience (1)		1	'Are you born in the today's area of Germany?' *Yes (1) / No (2) / Don't know (3) / No information (4)*
			1	

	Semester hours and Semester hours with mandatory attendance (2)		2	'How many semester hours per week do you take this semester? Thereof, how many with obligation to attend?'
	Field of study (1)		1	Grouped variable created of: 'What is your current study-subject or special-subject?' *Drop-down list of all subjects of the university of Mainz*
	First year (Semester/Study progress) (1)		1	'What is the number of your current university semester?'
**Psychological variables**	Somatic symptoms (1)	SSS-8[1]	8	
	Depressive symptoms (1)	PHQ-9[2]	9	
	Symptoms of general anxiety (1)	GAD-2[3]	2	
	Loneliness (1)	UCLA[3]	3	
	Emotional Exhaustion (1)	MBI-SS[4]	5	
	Social Anxiety (1)	Mini-SPIN[5]	3	
**Study-related psychosocial variables**	Presenteeism (1)	Töpritz et al.[6]	1	
	Absenteeism (1)	Töpritz et al.[6]	1	
	Planning competence and Competence for self-motivation (2)	Dettmers & Claußß[7]	8	
	Excessive demand (qualitative) (1)	BARI-S[8]	2	
	Competition (1)	Fend[9]	4	
	Cognitive irritation (1)	Mohr et al.[10]		
	Self-efficacy expectancy (1)	Self-Efficacy Scale; Adapted from Rigotti et al.[11]	6	
	Procrastination (1)	GPS-K[12]	9	
	Self-endangering behavior (1)	Krause et al.[13]	4	
	Performance pressure (1)	Fend[9]	5	
	Self-perceived Employability (1)	Self-constructed	1	
	Autonomy (1)	BARI-S[8]	6	

	Coping via use of: configuration options and social support (2)	In style of Daniels et al.[14]	4	
	Social support of: fellow students and lecturers (2)	SALSA[15]	3	
**General psychosocial variables**	Self-criticism (1)	DEQ-SC4[16]; Tibubos et al.[17]	4	
	Self-acceptance (1)	Ryff[18]; Ryff & Keyes[19]	3	
	Emotion regulation, emotion suppression, and impulsiveness (3)	ERQ[20]	4	
**Health behavior variables**	Healthy diet (1)	Self-constructed	1	'Healthy diet consists of eating plentiful whole grains, potatoes, fruit and vegetable several times a day, food low in trans fats, little red meat and sausage, little fast-food (e.g. burger, fries), little sugar-rich food (e.g. lemonade, soft drinks, candy, cake). To what extend did you stick to a healthy diet in the last month?' *Not at all (1) – Very strong (5)*
	Moderate-vigorous physical activity (min/day) and Sedentary Behavior (2)	IPAQ short version[21]	7	
	Alcohol use (1)	AUDIT-C (Cut-Off: 4)[22]	3	
	Smoking cigarettes (1)	Töpritz et al.[6]	1	
	Coffee, Energy drinks, Caffeine tablets, Cola, Ginkgo biloba (5)	Dietz et al.[23]	1	
	Social media use (1)	Adapted from Stark et al.[24]	6	
	Fruit and Vegetable Consumption (2)	European Commission, 2013[25]; Fehr et al.[26]	4	

Supplementary table 1. List of independent variables differentiated in the five category groups. 'Count' means the count of variables if 2 or more variables of the same scale are listed together.

References

1. Gierk, B. *et al.* The somatic symptom scale-8 (SSS-8): a brief measure of somatic symptom burden. *JAMA internal medicine* **174,** 399–407; 10.1001/jamainternmed.2013.12179 (2014).
2. Kroenke, K., Spitzer, R. L. & Williams, J. B. The PHQ-9: validity of a brief depression severity measure. *Journal of general internal medicine* **16,** 606–613; 10.1046/j.1525-1497.2001.016009606.x (2001).
3. Döring, N., & Bortz, J. Psychometrische Einsamkeitsforschung: Deutsche Neukonstruktion der UCLA Loneliness Scale [Psychometric research on loneliness: A new German version of the University of California at Los Angeles (UCLA) Loneliness Scale]. *Diagnostica* **39,** 224–239 (1993).
4. Gumz, A., Erices, R., Brähler, E. & Zenger, M. Faktorstruktur und Gütekriterien der deutschen Übersetzung des Maslach-Burnout-Inventars für Studierende von Schaufeli et al. (MBI-SS). *Psychotherapie, Psychosomatik, medizinische Psychologie* **63,** 77–84; 10.1055/s-0032-1323695 (2013).
5. Wiltink, J. *et al.* Mini - social phobia inventory (mini-SPIN): psychometric properties and population based norms of the German version. *BMC psychiatry* **17,** 377; 10.1186/s12888-017-1545-2 (2017).
6. Töpritz, K. *et al. Wie gesund sind Studierende der Technischen Universität Kaiserslautern? Ergebnisse der Befragung 06/15* (Schriftenreihe des AB Public Health: Prävention und psychosoziale Gesundheitsforschung: Nr. 01/P16, Berlin: Freie Universität Berlin., 2016).
7. Dettmers, J. & Clauß, E. Arbeitsgestaltungskompetenzen für flexible und selbstgestaltete Arbeitsbedingungen. In *Gestaltungskompetenzen für gesundes Arbeiten,* edited by M. Janneck & A. Hoppe (Springer Berlin Heidelberg, Berlin, Heidelberg, 2018), pp. 13–25.
8. Gusy, B. & Lohmann, K. Berliner-Anforderungs Ressourcen Inventar für Studierende. In *Instrumentenhandbuch zum Projekt, University Health Report,* edited by B. Gusy & K. Lohmann (Schriftenreihe des Instituts für Prävention und psychosoziale Gesundheitsforschung (Nr 01/P14), Berlin: Freie Universität Berlin, 2014), pp. 31–40.
9. Fend, H. *Der Umgang mit der Schule in der Adoleszenz [Coping with school during adolescence]* (Huber, Bern, 1997).
10. Mohr, G., Rigotti, T. & Müller, A. Irritation - ein Instrument zur Erfassung psychischer Beanspruchung im Arbeitskontext. Skalen- und Itemparameter aus 15 Studien. *Zeitschrift für Arbeits- und Organisationspsychologie A&O* **49,** 44–48; 10.1026/0932-4089.49.1.44 (2005).
11. Rigotti, T., Schyns, B. & Mohr, G. A Short Version of the Occupational Self-Efficacy Scale: Structural and Construct Validity Across Five Countries. *Journal of Career Assessment* **16,** 238–255; 10.1177/1069072707305763 (2008).
12. Klingsieck, K. B. & Fries, S. Allgemeine Prokrastination. *Diagnostica* **58,** 182–193; 10.1026/0012-1924/a000060 (2012).
13. Krause, A. *et al.* Selbstgefährdung als Indikator für Mängel bei der Gestaltung mobil-flexibler Arbeit: Zur Entwicklung eines Erhebungsinstruments. *Wirtschaftspsychologie* (2015).
14. Daniels, K., Beesley, N., Cheyne, A. & Wimalasiri, V. Coping processes linking the demands-control-support model, affect and risky decisions at work. *Human Relations* **61,** 845–874; 10.1177/0018726708093543 (2008).
15. Rimann, M., & Udris, I. Subjektive Arbeitsanalyse: Der Fragebogen SALSA. In *Unternehmen arbeitspsychologisch bewerten. Ein Mehr-Ebenen-Ansatz unter besonderer Berücksichtigung von Mensch, Technik und Organisation,* edited by O. Strohm, & E. Ulich (vdf Hochschulverlag, Zürich, Switzerland, 1997), pp. 281–298.

16. Rudich, Z., Lerman, S. F., Gurevich, B., Weksler, N. & Shahar, G. Patients' self-criticism is a stronger predictor of physician's evaluation of prognosis than pain diagnosis or severity in chronic pain patients. *The journal of pain : official journal of the American Pain Society* **9,** 210–216; 10.1016/j.jpain.2007.10.013 (2008).

17. Tibubos, A. N. *et al.* Trait self-criticism in the general population: German normative data and psychometric properties of the Depressive Experiences Questionnaire Self-Criticism 4 (DEQ-SC4). *Journal of Personality Assessment* (2021, submitted).

18. Ryff, C. D. Happiness is everything, or is it? Explorations on the meaning of psychological well-being. *Journal of Personality and Social Psychology* **57,** 1069 (1989).

19. Ryff, C. D. & Keyes, C. L. The structure of psychological well-being revisited **69,** 719–727 (1995).

20. Abler, B. & Kessler, H. ERQ - Emotion Regulation Questionnaire - deutsche Fassung. In *Elektronisches Testarchiv,* edited by Leibniz-Zentrum für Psychologische Information und Dokumentation (ZPID) (ZPID, Trier, 2011).

21. Craig, C. L. *et al.* International physical activity questionnaire: 12-country reliability and validity. *Medicine and science in sports and exercise* **35,** 1381–1395; 10.1249/01.MSS.0000078924.61453.FB (2003).

22. Bush. The AUDIT Alcohol Consumption Questions (AUDIT-C): An Effective Brief Screening Test for Problem Drinking (1998).

23. Dietz, P., Soyka, M. & Franke, A. G. Pharmacological Neuroenhancement in the Field of Economics-Poll Results from an Online Survey. *Frontiers in psychology* **7,** 520; 10.3389/fpsyg.2016.00520 (2016).

24. Stark, B., Magin, M. & Jürgens, P. *Ganz meine Meinung? Informationsintermediäre und Meinungsbildung - eine Mehrmethodenstudie am Beispiel von Facebook* (Landesanstalt für Medien Nordrhein-Westfalen (LfM), Düsseldorf, 2017).

25. European Commission. *European Health Interview Survey (EHIS wave 2). Methodological manual : 2013 edition* (Publications Office of the European Union, Luxembourg, 2013).

26. Fehr, A., Lange, C., Fuchs, J., Neuhauser, H. & Schmitz, R. Gesundheitsmonitoring und Gesundheitsindikatoren in Europa. *Journal of Health Monitoring* **2**; 10.17886/RKI-GBE-2017-004.2 (2017).

				Correlation coefficient (Spearman)																																					
				p																																					
		M	SD	1	2	3	4	5	6	7	8	9	10	11	12	13	14	15	16	17	18	19	20	21	22	23	24	25	26	27	28	29	30	31	32	33	34	35	36	37	38
1	Pharmacological neuroenhancement	-	-	-
2	Age	23.76	4.35	.002	-
3	Financial status	773.96	520.39	.047**	.362***	-
4	Semester hours	17.23	10.67	-.031	-.250***	-.096***	-
5	Semester hours with mandatory attendance	8.53	8.09	-.044**	-.100***	-.049**	.538***	-
6	**Somatic symptoms**	8.43	5.13	.117***	-.062***	-.089***	.005	-.014	-
7	**Depressive symptoms**	7.41	5.16	.165***	-.046**	-.111***	-.012	-.030	.674***	-
8	**General anxiety**	1.97	1.63	.106***	-.067***	-.091***	-.004	-.002	.519***	.668***	-
9	Social anxiety	2.91	2.36	.045**	-.052***	-.129***	-.007	-.025	.303***	.428***	.395***	-
10	**Loneliness**	3.86	2.69	.078***	-.040**	-.110***	-.006	-.005	.367***	.529***	.466***	.450***	-
11	**Emotional exhaustion**	3.78	1.38	.102***	-.015	-.067***	.145***	.040*	.462***	.541***	.435***	.246***	.328***	-
12	Fruits per day	1.62	1.25	-.015	.014	.018	.033	.022	-.054***	-.054***	-.020	-,079***	-.048**	-.046**	-
13	Vegetables per day	1.97	2.15	.014	.045**	.087***	.002	.006	-.048**	-.042**	-.031	-.045**	-.028	-.033*	.353***	-
14	**Moderate-vigorous physical activity (minutes/day)**	125.54	101.31	.060***	-.010	-.004	.020	.039*	-.028	-.077***	-.074***	-.068***	-.093***	-.045**	.104***	-.079***	-
15	Sedentary behavior (hours/day)	7.43	2.74	-.034*	.019	.037*	.010	-.036*	.097***	.121***	.118***	.052**	.112***	-.124***	-.058***	-.001	-.242***	-
16	**Presenteeism**	5.19	10.95	.088***	.057***	-.012	.088***	.068***	.386***	.331***	.275***	.097***	.155***	.397***	.002	-.002	.051**	.034*	-
17	**Absenteeism**	1.90	5.37	.113***	.000	-.001	-.022	-.047**	.231***	.173***	.147***	.073***	.091***	.124***	-.034*	-.022	.001	-.010	.265***	-
18	Self-perceived employability	4.93	1.41	-.050**	.113***	.205***	.191***	.098***	-.239***	-.305***	-.259***	-.244***	-.232***	-.151***	.073***	.058***	.047**	.005	-.088***	-.145***	-
19	Autonomy	2.40	.81	-.009	.014	-.022	-.296***	-.157***	-.061***	-.071***	-.057***	-.001	-.045**	-.321***	-.034*	-.051**	.047**	-.071***	-.151***	.041*	-.156***	-
20	Coping via use of configuration options	1.73	.89	.051**	.056***	.019	-.122***	-.052***	.062***	.061***	.087***	.072***	.056***	-.062***	-.003	.009	.043**	-.044**	.027	.101***	-.109***	.408***	-
21	Coping via use of social support	1.97	.99	.023	.022	.047**	.041*	.059***	.099***	.078***	.129***	.028	.058***	.080***	.010	.042**	-.001	.014	.118***	.049**	.036*	.043**	.297***	-
22	Self-endangering behavior	3.21	.88	.043**	.054***	-.012	.203***	.132***	.304***	.298***	.260***	.085***	.145***	.499***	.030	.016	-.014	.117***	.435***	.022	.038*	-.274***	-.039*	.117***	-
23	**Planning competency**	3.58	.72	-.105***	-.006	.053***	.046**	.112***	-.239***	-.363***	-.255***	-.240***	-.237***	-.314***	.095***	.085***	.075***	-.051**	-.116***	-.141***	.280***	-.086***	-.005	-.036*	-.053***	-
24	**Excessive demands**	2.73	1.10	.063***	-.094***	-.109***	.170***	.002	.228***	.278***	.225***	.155***	.169***	.494***	-.017	-.047**	-.031	.062***	.224***	.041*	-.062***	-.251***	-.059***	.088***	.274***	-.296***	-
25	**Competition**	1.74	.68	.056***	.056***	.025	.075***	-.081***	.142***	.128***	.119***	.092***	.102***	.260***	.018	.002	.050**	.007	.183***	.052***	-.029	-.185***	-.002	.014	.183***	-.070***	.181***	-
26	Performance pressure	3.19	.96	.027	.001	.004	.344***	.114***	.156***	.149***	.141***	.034*	.072***	.506***	.043*	.025	-.005	.097***	.317***	.000	.211***	-.471***	-.161***	.098***	.500***	-.073***	,480***	.294***	-
27	**Cognitive irritation**	3.88	1.64	.087***	.018	-.056***	.084***	.029	.394***	.470***	.445***	.255***	.301***	.621***	-.011	-.007	-.055***	.116***	.385***	.068***	-.134***	-.213***	.002	.132***	.497***	-.240***	.372***	.233***	.437***	-
28	Social support of lectures	2.89	.91	-.042*	-.020	-.055***	-.117***	.013	-.137***	-.156***	-.121***	-.058***	-.106***	-.348***	-.010	-.042*	.032*	-.037*	-.213***	-.020	-.010	.443***	.169***	.092***	-.247***	.116***	-.245***	-.302***	-.351***	-.243***	-
29	**Social support of fellow students**	3.69	.90	-.082***	-.076***	.032*	.093***	.096***	-.151***	-.225***	-.158***	-.178***	-.242***	-.188***	.008	.049**	.040*	-.004	-.082***	-.096***	.234***	.027	.001	.208***	-.011	.201***	-.087***	-.324***	-.004	-.139***	.256***	-
30	**Competence for self-motivation**	3.16	.75	-.097***	.034*	.074***	.077***	.098***	-.322***	-.478***	-.334***	-.281***	-.304***	-.462***	.117***	.078***	.105***	-.083***	-.131***	-.144***	.321***	.147***	.033*	.012	-.068***	.566***	-.316***	-.098***	-.061***	-.298***	.214***	.249***	-
31	**Self-efficacy expectancy**	3.29	.77	-.071***	.082***	.152***	-.013	.046**	-.357***	-.490***	-.413***	-.313***	-.328***	-.485***	.064***	.067***	.106***	-.084***	-.222***	-.103***	.403***	.211***	.063***	-.027	-.197***	.536***	-.489***	-.131***	-.200***	-.413***	.267***	.232***	.599***	-
32	**Procrastination**	23.05	6.05	.118***	.022	-.015	-.078***	-.062***	.186***	.302***	.159***	.187***	.185***	.184***	-.147***	-.082***	-.087***	.059***	-.004	.171***	-.209***	.025	.013	.001	-.032*	-.561***	.091***	-.043**	-.085***	.063***	-.001	-.108***	-.481***	-.271***	-
33	**Self-criticism**	18.66	4.73	.064***	-.009	-.028	-.002	-.035*	.313***	.477***	.419***	.314***	.383***	.393***	.005	.013	-.072***	.084***	.200***	.065***	-.193***	-.112***	-.006	.016	.264***	-.264***	.183***	.145***	.167***	.410***	-.159***	-.145***	-.331***	-.380***	.179***	-
34	**Self-acceptance**	10.73	2.92	-.088***	-.039*	.108***	.070***	.078***	-.328***	-.542***	-.411***	-.414***	-.436***	-.347***	.054***	.050**	.101***	-.080***	-.157***	-.127***	.412***	.048**	-.018	.026	-.146***	.398***	-.180***	-.110***	-.013	-.306***	.122***	.263***	.468***	.512***	-.304***	-.514***	-
35	Emotion regulation	9.25	2.62	-.021	,040*	.047**	-.012	.005	-.130***	-.208***	-.161***	-.126***	-.145***	-.155***	.075***	.092***	.043**	-.058***	-.046**	-.010	.141***	.036*	.066***	.080***	-.056***	.183***	-.110***	-.065***	-.018	-.103***	.078***	.161***	.239***	.277***	-.119***	-.148***	.285***	-
36	Emotion suppression	8.28	3.24	.032	-.035*	-.104***	.041*	-.016	.062***	.181***	.091***	.222***	-.205***	.092***	-.030	-.053***	-.010	.051**	.042**	-.020	-.088***	-.013	-.041*	-.141***	.064***	-.088***	.043**	.022	.037*	.085***	-.033*	-.154***	-.120***	-.089***	.095***	.177***	-.281***	-.153***	-
37	**Impulsiveness**	4.50	2.05	.068***	.021	.057***	-.022	.022	.056***	.023	-.022	-.085***	-.050**	.033*	.014	.023	.061***	-.040*	.059***	.063***	.002	.014	.045**	.050**	-.004	-.050**	.037*	.053***	.001	-.027	.021	-.001	-.025	-.008	.097***	-.035*	.024	.017	-.200***	-
38	**Social media use**	1.94	1.42	.054***	-.229***	-.051***	.033*	.020	.100***	.044**	.049***	.025	-.006	.054***	-.005	-.067***	.105***	-.080***	.042**	.029	-.061***	-.010	-.016	-.013	.000	.019	.086***	.091***	-.063***	.009	-.030	.029	-.037*	-.073***	.037*	.035*	.018	-.028	-.065***	.088***	-

Supplementary Table 2. Bivariate correlations, means, and standard deviations of continuous study variables. Range of observed cases: n = 3,440-4,328; M = mean; SD = standard deviation; ***: $p \leq .001$; **: $p \leq .01$; *: $p \leq .05$.; **variables in bold**: significant ($p \leq .001$) associations with pharmacological neuroenhancement

	**Association with the 12-month prevalence of PN**	
	χ^2	*p*
Gender (*n* = 3,800)	(2) = 18.802	≤ .001
Degree (*n* = 3,800)	(7) = 25.169	.001
Relationship status (*n* = 3,796)	(2) = 3.325	.190
Employment (*n* = 3,800)	(3) = 8.260	.041
Support by parents/relatives (*n* = 3,785)	(1) = .675	.411
Parent's educational background (*n* = 3,798)	(8) = 9.735	.284
Migration background (*n* = 3,799)	(3) = 10.276	.016
Personal migration experience (*n* = 3,799)	(3) = 6.484	.090
Field of study (*n* = 3,791)	(6) = 10.337	.111
First year (*n* = 3,700)	(1) = 2.284	.131
Healthy diet (*n* = 3,800)	(4) = 22.792	≤ .001
Alcohol use (AUDIT-C Cut-off: 4) (*n* = 3,796)	(1) = 73.920	≤ .001
Smoking cigarettes (*n* = 3,800)	(4) = 262.566	≤ .001
Coffee (*n* = 3,800)	(3) = 79.112	≤ .001
Energy drinks (*n* = 3,800)	(3) = 99.658	≤ .001
Caffeine tablets (*n* = 3,800)	(3) = 151.702	≤ .001
Cola (*n* = 3,800)	(3) = 80.497	≤ .001
Ginkgo biloba (*n* = 3,799)	(3) = 40.141	≤ .001

Supplementary table 3. Relationship to the 12-month prevalence of pharmacological neuroenhancement of all selected categorial study variables. χ^2: Pearson Chi-square value

12-month use of PN

		Step 1: sociodemographic variables (R^2 = 0.01***)	Step 2: psychological variables (R^2 = 0.071***)	Step 3: study-related psychosocial variables (R^2 = 0.092***)	Step 4: general psychosocial variables (R^2 = 0.1***)	Step 5: health-behavioral variables (R^2 = 0.257***)
		**OR (95% CI)**	**OR (95% CI)**	**OR (95% CI)**	**OR (95% CI)**	**OR (95% CI)**
**Sociodemographic variables**						
Gender	Female	Reference***	Reference***	Reference***	Reference	Reference
	Male	1.517 (1.214 – 1.897)***	1.764 (1.414 – 2.277)***	1.695 (1.328 – 2.163)***	1.768 (1.380 – 2.265)***	1.387 (1.052 – 1.827)*
	Diverse	3.190 (1.342 – 7.580)**	2.183 (0.877 – 5.431)	2.296 (0.919 – 5.736)	2.128 (0.842 – 5.377)	1.780 (0.638 – 4.971)
**Psychological variables**						
Somatic symptoms			1.019 (0.991 – 1.048)	1.013 (0.985 – 1.042)	1.011 (0.983 – 1.040)	0.993 (0.962 – 1.024)
Depressive symptoms			1.102 (1.067 – 1.139)***	1.091 (1.054 – 1.130)***	1.095 (1.056 – 1.135)***	1.086 (1.044 – 1.129)***
General anxiety			0.987 (0.902 – 1.079)	1.009 (0.920 – 1.107)	1.021 (0.930 – 1.120)	1.025 (0.926 – 1.134)
Loneliness			0.968 (0.924 – 1.013)	0.954 (0.910 – 1.000)*	0.965 (0.920 – 1.013)	0.999 (0.948 – 1.052)
Emotional exhaustion			1.031 (0.940 – 1.132)	0.962 (0.854 – 1.084)	0.961 (0.853 – 1.083)	0.966 (0.849 – 1.101)
**Study-related psychosocial variables**						
Presenteeism				1.005 (0.996 – 1.014)	1.006 (0.997 – 1.014)	1.005 (0.996 – 1.015)
Absenteeism				1.019 (1.004 – 1.035)*	1.019 (1.003 – 1.034)*	1.022 (1.005 – 1.040)**
Planning competency				0.896 (0.728 – 1.104)	0.898 (0.729 – 1.107)	0.894 (0.712 – 1.122)
Excessive demands				1.103 (0.979 – 1.244)	1.082 (0.959 – 1.222)	1.106 (0.969 – 1.263)

Competition		1.084 (0.914 – 1.287)	1.072 (0.902 – 1.275)	0.962 (0.796 – 1.262)
Cognitive irritation		1.057 (0.966 – 1.156)	1.080 (0.985 – 1.183)	1.081 (0.980 – 1.192)
Social support (fellow students)		0.853 (0.749 – 0.970)*	0.856 (0.752 – 0.975)*	0.830 (0.721 – 0.955)**
Competence for self-motivation		1.023 (0.827 – 1.265)	1.005 (0.812 – 1.244)	0.999 (0.795 – 1.256)
Self-efficacy expectancy		1.304 (1.055 – 1.611)*	1.258 (1.010 – 1.567)*	1.230 (0.968 – 1.563)
Procrastination		1.031 (1.008 – 1.056)**	1.028 (1.010 – 1.567)*	1.013 (0.987 – 1.039)
**General psychosocial variables**				
Self-criticism			0.975 (0.947 – 1.004)	0.955 (0.926 – 0.986)**
Self-acceptance			1.004 (0.954 – 1.057)	0.980 (0.927 – 1.035)
Impulsiveness			1.096 (1.041 – 1.155)***	1.027 (0.971 – 1.087)
**Health behavior variables**				
Healthy diet				1.264 (1.117 – 1.431)***
Moderate-vigorous physical activity (min/day)				1.001 (1.000 – 1.002)*
Alcohol use				1.612 (1.243 – 2.092)***
Smoking cigarettes				1.468 (1.349 – 1.597)***
Coffee	never (n = 1284)			Reference
	within the last 30 days (n = 1766)			1.531 (1.114 – 2.106)**

	within the last 12 months (*n* = 387)	1.135 (0.719 – 1.793)
	more than 12 months ago (*n* = 147)	0.904 (0.425 – 1.926)
Energy drinks	never (*n* = 2354)	Reference
	within the last 30 days (*n* = 553)	1.166 (0.844 – 1.612)
	within the last 12 months (*n* = 374)	1.328 (0.932 – 1.891)
	more than 12 months ago (*n* = 303)	0.864 (0.557 – 1.340)
Caffeine tablets	never (*n* = 3222)	Reference***
	within the last 30 days (*n* = 91)	4.235 (2.552 – 7.027)***
	within the last 12 months (*n* = 85)	1.763 (1.011 – 3.073)*
	more than 12 months ago (*n* = 186)	2.117 (1.416 – 3.164)***
Coke	never (*n* = 1889)	Reference***
	within the last 30 days (*n* = 1106)	1.669 (1.255 – 2.221)***
	within the last 12 months (*n* = 453)	2.267 (1.587 – 3.239)***
	more than 12 months ago (*n* = 136)	1.256 (0.625 – 2.524)
Ginkgo biloba	never (*n* = 3451)	Reference
	within the last 30 days (*n* = 37)	2.587 (1.164 – 5.747)*
	within the last 12 months (*n* = 32)	1.095 (0.442 – 2.711)

more than 12 months ago (n = 64)	1.417 (0.697 – 2.881)
Social media use	0.997 (0.913 – 1.088)

Supplementary table 4. Binary logistic regression analysis to predict the dependent variable '12-month prevalence of pharmacological neuroenhancement' by stepwise inclusion of the 5 independent variable groups. *O*bserved cases: n = 3,608; ***: $p \leq .001$; **: $p \leq .01$; *: $p \leq .05$; Step 1: χ^2 (2) = 17.504; Step 2: χ^2 (7) = 129.270; Step 3: χ^2 (17) = 167.444; Step 4: χ^2 (20) = 183.304; Step 5: χ^2 (40) = 490.590

12-month use of PN

		Step 5: health-behavioral variables (R^2 = 0.254***)
		**OR (95% CI)**
**Sociodemographic variables**		
Gender	Female	Reference
	Male	1.451 (1.064 – 1.979)**
	Diverse	2.910 (0.997 – 8.494)
**Psychological variables**		
Somatic symptoms		1.005 (0.971 – 1.041)
Depressive symptoms		1.075 (1.029 – 1.122)***
General anxiety		1.016 (0.907 – 1.139)
Loneliness		1.009 (0.951 – 1.071)
Emotional exhaustion		0.960 (0.830 – 1.111)
**Study-related psychosocial variables**		
Presenteeism		1.005 (0.995 – 1.016)
Absenteeism		1.026 (1.007 – 1.045)**
Planning competency		0.868 (0.672 – 1.121)
Excessive demands		1.089 (0.938 – 1.266)
Competition		1.049 (0.848 – 1.297)
Cognitive irritation		1.056 (0.946 – 1.179)
Social support (fellow students)		0.900 (0.768 – 1.055)
Competence for self-motivation		1.015 (0.784 – 1.315)
Self-efficacy expectancy		1.180 (0.898 – 1.551)
Procrastination		1.008 (0.979 – 1.037)
**General psychosocial variables**		
Self-criticism		0.966 (0.932 – 1.001)
Self-acceptance		0.978 (0.919 – 1.040)

Impulsiveness		1.045 (0.980 – 1.114)
**Health behavior variables**		
Healthy diet		1.219 (1.061 – 1.400)**
Moderate-vigorous physical activity (min/day)		1.001 (1.000 – 1.003)*
Alcohol use		1.463 (1.094 – 1.995)**
Smoking cigarettes		1.502 (1.366 – 1.651)***
Coffee	never (*n* = 1284)	Reference
	within the last 30 days (*n* = 1766)	1.463 (1.017 – 2.103)*
	within the last 12 months (*n* = 387)	1.107 (0.659 – 1.860)
	more than 12 months ago (*n* = 147)	0.867 (0.366 – 2.053)
Energy drinks	never (*n* = 2354)	Reference
	within the last 30 days (*n* = 553)	1.101 (0.760 – 1.595)
	within the last 12 months (*n* = 374)	1.370 (0.920 – 2.041)
	more than 12 months ago (*n* = 303)	0.915 (0.557 – 1.500)
Caffeine tablets	never (*n* = 3222)	Reference***
	within the last 30 days (*n* = 91)	3.934 (2.245 – 6.891)***
	within the last 12 months (*n* = 85)	1.214 (0.620 – 2.375)
	more than 12 months ago (*n* = 186)	2.365 (1.527 – 3.663)***
Coke	never (*n* = 1889)	Reference***
	within the last 30 days (*n* = 1106)	1.774 (1.282 – 2.455)***
	within the last 12 months (*n* = 453)	2.477 (1.654 – 3.710)***
	more than 12 months ago (*n* = 136)	1.064 (0.471 – 2.401)
Ginkgo biloba	never (*n* = 3451)	Reference
	within the last 30 days (*n* = 37)	2.462 (1.058 – 5.733)*
	within the last 12 months (*n* = 32)	0.869 (0.316 – 2.540)
	more than 12 months ago (*n* = 64)	1.286 (0.579 – 2.857)
Social media use		0.967 (0.877 – 1.067)

Supplementary table 5. Cross-validation of the binary logistic regression analysis to predict the dependent variable ‘12-month prevalence of pharmacological neuroenhancement’ using an 80% random sample. Observed cases: n = 2,889; ***: $p \leq .001$; **: $p \leq .01$; *: $p \leq .05$; Step 5: χ^2 (40) = 385.123; correctly classified cases: 89.6%

	Past 12 months use of PN	
	**No**	**Yes**
**Predictors**		
Gender – male	81.4% (*n* = 903)	13.2% (*n* = 146)
Reference: female	86.5% (*n* = 2458)	9.3% (*n* = 263)
Depressive symptoms (score: 0 – 27), mean ± SD	7.0 ± 4.9 (*n* = 3380)	9.9 ± 5.8 (*n* = 416)
Absenteeism (number in days), mean ± SD	1.7 ± 4.5 (*n* = 3314)	3.4 ± 9.0 (*n* = 403)
Social support by fellow students, mean ± SD (score: 1 – 5)	3.7 ± 0.9 (*n* = 3316)	3.5 ± 1.0 (*n* = 404)
Self-criticism, mean ± SD (score: 7 – 28)	18.6 ± 4.7 (*n* = 3305)	19.4 ± 5.1 (*n* = 398)
Healthy diet, mean ± SD (score: 1 – 5)	3.3 ± 1.0 (*n* = 3384)	3.4 ± 1.1 (*n* = 416)
Moderate-vigorous physical activity (min/day), mean ± SD	122.9 ± 98.8 (*n* = 3333)	145.7 ± 119.2 (*n* = 408)
Risky alcohol use (*n* = 1634)	40.6% (*n* = 1373 of 3380)	62.7% (*n* = 261 of 416)
Smoking cigarettes currently (*n* = 542)	11.5% (*n* = 388 of 3384)	37.0% (*n* = 154 of 416)
**Soft neuroenhancers:**		
Coffee – never (reference)	94.5% (*n* = 1,288)	5.5% (*n* = 75)
Coffee – within the last 30 days	84.8% (*n* = 1,590)	15.2% (*n* = 284)
Caffeine tablets – never (reference)	91.1% (*n* = 3,105)	8.9% (*n* = 304)
Caffeine tablets – within the last 30 days	63.0% (*n* = 63)	37.0% (*n* = 37)
Caffeine tablets – more than 12 month ago	76.0% (*n* = 152)	24.0% (*n* = 48)
Coke – never (reference)	93.2% (*n* = 1,867)	6,8% (*n* = 137)
Coke – within the last 30 days	84,1% (*n* = 990)	15,9% (*n* = 187)
Coke – within the last 12 month	83.4% (*n* = 396)	16.6% (*n* = 79)
Ginkgo biloba – never (reference)	89.7% (*n* = 3,279)	10.3% (*n* = 378)
Ginkgo biloba – within the last 30 days	64.1% (*n* = 25)	35.9% (*n* = 14)

Supplementary table 6. Descriptive statistics of the predictors of PN among survey participants (*n* = 3,984). SD: standard deviation
